# Modelling EAT-lancet dietary patterns scenarios: effects on environmental and health metrics in children and adolescents

**DOI:** 10.1007/s00394-026-04069-6

**Published:** 2026-07-21

**Authors:** Beatriz Teixeira, Cláudia Afonso, Catarina Carvalho, Duarte Torres, Carla Lopes, Milton Severo, Andreia Oliveira

**Affiliations:** 1 Faculty of Nutrition and Food Sciences, University of Porto, Rua do Campo Alegre, 823, 4150-180 Porto, Portugal; 2 EPIUnit ITR, Institute of Public Health of the University Porto, University of Porto, Rua das Taipas 135, 4050-600 Porto, Portugal; 3 School of Medicine and Biomedical Sciences, Instituto de Ciências Biomédicas Abel Salazar, University of Porto, Rua Jorge de Viterbo Ferreira 228, 4050-313 Porto, Portugal; 4 Faculty of Medicine, University of Porto, Alameda Prof. Hernâni Monteiro, 4200-319 Porto, Portugal

**Keywords:** Childhood, Adolescence, Diet, Health, Sustainability

## Abstract

**Purpose:**

To simulate realistic dietary scenarios aligned with the EAT-Lancet reference diet and evaluate their impact on diet-related greenhouse gas emissions (GHGE), land use (LU), and obesity status in paediatric age.

**Methods:**

Dietary intake of children (3–9 y) and adolescents (10–17 y) from the last National Food, Nutrition, and Physical Activity Survey, Portugal (n = 1153), was assessed using food diaries (children) and 24-h recalls (adolescents) and an automated multiple-pass method with portion size estimations. The World Index for Sustainability and Health (WISH) measured adherence to the EAT-Lancet reference diet, reflecting dietary quality aligned with planetary health principles. Diet-related environmental indicators (greenhouse gas emissions [GHGE] and land use [LU]) were estimated using the SHARP-Indicators database. BMI (measured) z-scores were classified according to WHO criteria. Dietary patterns (DP) were derived using latent class analysis. Simulations aligned current dietary intake with the EAT-Lancet reference diet. The potential to prevent obesity cases was estimated by Potential Impact Fractions (PIF).

**Results:**

Overall, current diets are not aligned with the Planetary Health Diet. Among children, reducing the consumption of eggs, meats, fish, and soft drinks improved WISH scores and decreased environmental indicators. For adolescents, the greatest reductions of these indicators were achieved by reducing dairy and red meat. The scenario that led to the largest significant reduction in obesity cases involved a shift towards a plant-based dietary pattern (PIF = 0.7% children, 0.2% adolescents), which also increased environmental impact (with mean GHGE increasing from 3.9 to 4.1 kg CO_2_-eq), as it replaced diets already low in animal products with a plant-based pattern of greater overall food intake.

**Conclusions:**

To effectively enhance adherence to the EAT-Lancet diet, promoting plant-based foods should be coupled with reductions in dairy, meat, soft drinks, and total dietary quantity.

**Supplementary Information:**

The online version contains supplementary material available at 10.1007/s00394-026-04069-6.

## Introduction

In the recent years, the importance of incorporating environmental sustainability into dietary practices has received growing attention [1]. The environmental impact of food production, particularly in terms of greenhouse gas emissions (GHGE), land use (LU), and water use, has led to increased advocacy for more sustainable food choices [1]. The literature suggests that fostering environmental awareness from an early age may help reduce ecological footprints and support the health of future generations [2], highlighting the potential benefits of encouraging sustainable dietary habits since early life.

The extent to which healthy dietary patterns fully align with environmental sustainability remains a complex topic [3, 4]. Generally, foods that promote better health outcomes exhibit a low environmental impact. This suggests that dietary changes aimed at decreasing the prevalence of non-communicable diseases could concurrently support environmental sustainability goals [5]. On the other hand, it is important to note that, for example, animal-based products are rich sources of micronutrients such as calcium, vitamin B12, and iron, which are crucial for child development. In contrast, their production is associated with significant environmental challenges, such as high GHGE and extensive water use [6]. This highlights that the relationship between health and environmental sustainability is not always straightforward, and achieving an optimal balance requires careful consideration of both dietary benefits and ecological risk impacts [3, 4].

The Eat-Lancet Commission has proposed a reference diet to balance health and sustainability [7]. These emphasise dietary patterns based on specific food groups that promote health and environmental sustainability while supporting nutritional adequacy. These recommendations advocate for increased consumption of plant-based foods, reduced intake of red and processed meat, and prioritization of sustainably produced foods [7]. Importantly, these recommendations have also been adapted for children and adolescents, acknowledging the need to address their specific nutritional requirements while fostering lifelong sustainable eating habits [8]. The growing environmental challenges posed by unsustainable food systems [7], highlight the importance of implementing balanced dietary strategies tailored to this age group.

Despite this growing recognition, research specifically examining the balance between dietary healthiness and environmental sustainability in children and adolescents remains limited. Existing studies in paediatric populations have largely focused on characterising the environmental footprint of current dietary patterns or comparing predefined dietary groups in terms of environmental indicators [8, 9]. Some scenario-based studies have modelled the nutritional and environmental consequences of shifting children's diets towards national food-based dietary guidelines or the EAT-Lancet reference diet [10], or used linear optimisation to design climate-friendly diets for adolescents [11]. However, these approaches have generally relied on a priori dietary patterns based on predefined food group targets. To our knowledge, this is the first study to use data-driven dietary patterns based on the EAT-Lancet food group recommended tresholds and simultaneously evaluate their impact on environmental sustainability indicators and obesity status in children and adolescents. Therefore, tis study aimed to simulate realistic, well-fitted dietary scenarios aligned with the EAT-Lancet reference diet and evaluate their impact on environmental sustainability indicators, namely diet-related GHGE and LU, and the obesity status of children and adolescents.

## Methods

### Study design and participants

**Fig. 1:**
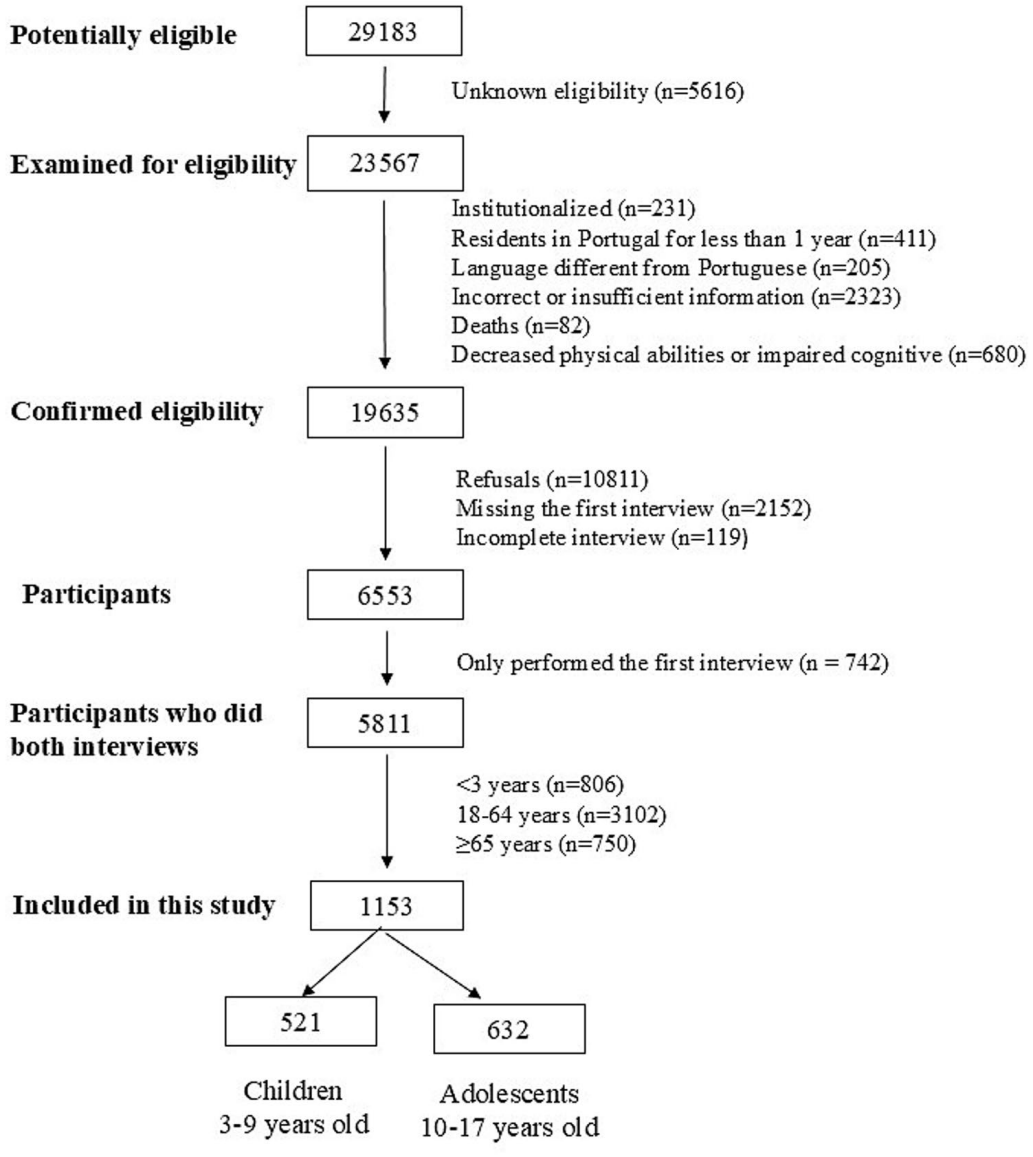
Flowchart of participants selection in this study

Participants are from the National Food, Nutrition, and Physical Activity Survey, Portugal (IAN-AF 2015–2016). This study recruited a representative sample of the general Portuguese population from 3 months to 84 years old from the National Health Registry via multistage sampling, with stratification based on sex, age groups, and geographical region [12]. In total, 5811 individuals completed two dietary assessment interviews conducted 8 to 15 days apart by trained researchers. Among them, 1153 were children (3–9 years, n = 521) and adolescents (10–17 years, n = 632), with participation proportions among eligible individuals at 47% and 46%, respectively. More information about the methodology of the participants’ selection is presented in Fig. 1.

Ethical approval was obtained from the National Commission for Data Protection (Authorisation number 4940/2015 from May 26, 2015), the Ethical Committee of the Institute of Public Health of the University of Porto (CE15033, from March 13, 2015), and the Ethical Commissions of each Regional Administration of Health. According to the Declaration of Helsinki, all participants were asked to provide their written informed consent. Written agreements from the main caregivers were required for children/adolescents. Adolescents were also asked to sign the consent form with their legal representative. All documents with identification data were treated separately and stored in a different dataset, ensuring data confidentiality.

### Procedures

#### Dietary data

Dietary intake was evaluated using two non-consecutive one-day food diaries for children, complemented by a face-to-face interview with the caregiver to obtain detailed descriptions and quantifications of the food consumed. For adolescents, two non-consecutive 24-h recalls were conducted; if the adolescents were between 10 and 14 years old, a caregiver was also required to be present [12]. The data from these 24-h recalls or food diaries were computerised in the ‘eAT24’ software (Electronic Assessment Tool for 24-h recalls). This software was previously validated [13]; it employs an automated multiple-pass method and includes food images for portion size estimations [14]. The software was previously validated against urinary biomarkers (nitrogen, potassium and sodium), showing acceptable validity for protein and potassium intake estimates, but lower agreement for sodium. All foods and beverages consumed over the 24 h were recorded for each eating occasion, quantified, and described as consumed. The average grams of foods and beverages were calculated from the two recorded days. Each food item was assigned a unique FoodEx2 classification code from EFSA [12].

An adapted version of the World Index for Sustainability and Health (WISH) [8] was applied to dietary data from children and adolescents to assess adherence to the EAT-Lancet reference diet. WISH assesses diet quality and environmental sustainability, encompassing 13 key food groups: (1) cereals; (2) vegetables; (3) fruits; (4) legume grains; (5) nuts; (6) unsaturated oils; (7) dairy; (8) fish and shellfish; (9) chicken and other poultry; (10) eggs; (11) red and processed meat; (12) saturated fats; (13) soft drinks and added sugars. According to the EAT-Lancet reference diet, each component is scored on a range from 0 to 10, indicating the extent to which dietary choices align with a healthy and environmentally sustainable diet, including positive, neutral, and penalized contributions depending on intake levels; full scoring rules are described in Supplementary Table A. The WISH is the sum of the scores across all food groups; the total score ranges from 0 to 130, with higher scores indicating a better diet from health and environmental sustainability perspectives [8]. This scoring system has been previously adapted and validated for the paediatric population [8].

#### Diet-related GHGE and LU

Using the SHARP-Indicators database (SHARP-ID), GHGE (in kg CO_2_eq) and LU (in m^2^/year) were computed to assess the environmental impact of each food group consumption. The SHARP-ID includes a life cycle inventory data of 944 food products used in Europe and coded with a specific FoodEx2-code from EFSA. Production, transportation, edible portions, cooking losses, and profits were all considered in the footprints. More details on SHARP-ID development can be found elsewhere [15].

Data from SHARP-ID was used to estimate the average environmental impact (GHGE and LU) of food groups, considering the WISH sore created. Individual diet-related GHGE (kg CO2-eq/day) and LU (m^2^·year/day) were subsequently estimated by combining the participant's daily consumption of each food group with the corresponding mean environmental impact value of that food group, linked through the FoodEx2 codes. For each participant, the environmental impact contributed by each food group was calculated as:

$$\frac{\begin{aligned} & {\mathrm{individual}}\;{\mathrm{food}}\;{\mathrm{group}}\;{\mathrm{consumption}}*{\mathrm{average}}\;{\mathrm{food}}\; \\ & \quad \; {\mathrm{group}}\;{\mathrm{environmental}}\;{\mathrm{impact}}\;({\mathrm{GHGE}}\;{\mathrm{and}}\;{\mathrm{LU}}\;{\mathrm{per}}100{\mathrm{g}}) \\ \end{aligned} }{{100}} $$

The total daily GHGE and LU were obtained by summing the contributions of all food groups.

#### Body Mass Index (BMI)

Trained research staff measured the weight and height of the participants following standardised protocols [16]. Weight was measured on a digital scale (SECA® 813, Hamburg, Germany) to the closest tenth of a kilogram. A portable wall stadiometer (SECA® 213) was used to measure participants’ heights to the nearest centimetre while standing, wearing light clothing, and barefoot. The child and adolescent’s age and sex-specific BMI z-score were determined according to the WHO criteria. For participants of the same age and sex, obesity was defined as a BMI for-age z-score > 2 SD (children older than 5 years of age) or > 3 SD (children less than 5 years of age) [17, 18].

#### Other variables

Data on the sex and age of participants, calculated as the difference between the initial interview date and their birth dates, were collected. The ages of the mother and father were documented at the time of the interview. Parental educational attainment was classified into three levels: “ ≤ 9th grade” (9 or less years of schooling), “10th–12th grade” (between 10 and 12 years of schooling) and “higher education” (more than 12 years of schooling) [12].

### Statistical analysis

Dietary patterns were first identified using latent class analysis based on the 13 food groups included in the WISH score. This approach allowed the classification of individuals into distinct dietary patterns according to their probability of consumption of each food group. Subsequently, simulations were conducted to model the prevalence of class membership aligned with the EAT-Lancet reference diet (adapted for paediatric populations) [8]. For each simulation, one of the 13 WISH food groups was modified at a time, aiming to approximate the corresponding EAT-Lancet target intake while remaining within the range of plausible estimates observed in the population. To achieve this, a data simulation process that preserves the observed correlations and dependencies between food items was applied.

The outputs of the 13 individual simulations were then compared against the baseline scenario in terms of WISH score, and levels of GHGE, and LU.

Further details of each step of the analytical framework are provided in the subsequent sections.

#### Definition of dietary patterns

Latent class analyses were performed to estimate the number of latent classes (dietary patterns) stratified by age (children and adolescents). These analyses considered the 13 food groups previously described as included in the WISH score [8]. Ten randomly selected starting values were used for each latent class model (one for children and another for adolescents) to ensure convergence to the optimal solution.

Food items were categorised into two or three categories based on the frequency of zeros: (a) if 90% or more of the values (of intake) were zeros, data were transformed into a binary format (1 for values greater than zero, 0 otherwise); (b) if 10% or fewer of the values were zeros, data were dichotomised at the median of non-zero values; and (c) for intermediate proportions (10% to 90%), data were categorised into three groups: nil; equal or below the sample median and above the sample median intake (among the consumers). These categorised food groups were then treated as ordered variables.

The Akaike Information Criterion (AIC) and the Bayesian Information Criterion (BIC) were calculated to estimate the optimal number of latent classes. One-, two-, three- and four-class solutions were tested [19]. The lowest scores of AIC and BIC indicate a better fit. Nevertheless, we also look for the interpretation of the dietary patterns derived to choose the final number of optimal latent classes (Supplementary Table B).

Energy intake was not constrained or included as an optimisation target. This approach was chosen to preserve naturally occurring dietary patterns and maintain realistic population-level consumption distributions.

#### Simulation process

To simulate data aligned with the EAT-Lancet reference diet (target values) adapted for paediatric populations [8], we conducted 13 simulations for children and 13 for adolescents. Each simulation focused on one of the 13 food groups previously described as included in the WISH score, aiming to achieve, as close as possible, the EAT-Lancet dietary consumption targets for that specific food group.

*Step 1—Calculation of the proportion of each class compared to the target*

A latent class model combined with an optimisation approach was conducted. An objective function was defined to minimise the squared deviation between the weighted mean of each food group considered and each target value while ensuring compliance with the specified constraints. The objective function was defined as follows:

$$ \left( {(p_{1} \times xs\left[ 1 \right]} \right. $$$$ + p_{2} \times xs\left[ 2 \right] $$$$ + p_{2} \times xs\left[ 2 \right] $$$$ + ... + p_{I} $$$$ \left. { \times xs\left[ I \right]} \right) $$$$ - target)^{2} $$, where $${p}_{i}(\mathrm{i}=\mathrm{1,2},3,..., \mathrm{I})$$ represents the probability of each latent class in the population, and $${xs}_{i}$$ denotes the mean value of the food group in the respective latent class. The "target" refers to each food group’s EAT-Lancet dietary consumption cut-off. This function aimed to minimise the difference between each food group’s modelled value and the EAT-Lancet target.

Specific constraints were applied: weights (pi) must sum to 1 and fall within the range [0.01, 0.99].

*Step 2—Synthetic Data Generation*

Synthetic data (p_1_, p_2,_ and p_3_) with the same sample size and the same number of food groups (n = 13) were generated using the “poLCA.simdata” function for both children and adolescents. The latent classes in this dataset were derived based on the categories described earlier in “Definition of dietary patterns” section

*Step 3—Definition of the continuous consumption values of the 13 food groups in the simulated data*

In the simulated data, the continuous consumption values for each of the 13 food groups were generated as follows: for a given food group category in the simulated data, a continuous value was randomly selected from the observed values from the same food category and the same latent class. This random selection was assigned to each simulated individual and its simulated food group category.

When performing the simulation for a specific food group, individuals were reassigned to the dietary pattern that best aligned with the EAT-Lancet dietary target for that food group. This reassignment reflects the principle that individuals adapt their dietary patterns to meet specific recommendations. As a result, changes in consumption for one food group led to shifts in the overall dietary profile because individuals moved between dietary patterns based on their optimized simulation.

To consolidate the simulations into representative scenarios, they were grouped based on two key criteria: the similarities in the proportion of individuals classified into each dietary pattern across the 13 simulations for each age group, alongside with a comparison between the simulated median values for key indicators—WISH score, diet-related GHGE, and LU—with these original values in the observed data. This methodological approach allowed the consolidation of 13 simulations into four scenarios for children and five scenarios for adolescents. The 13 simulation outputs were not combined through statistical aggregation. Instead, they were compared and grouped based on similarity in the distribution of latent classes and in key summary indicators (WISH score, GHGE, and LU), allowing the identification of coherent and stable dietary scenarios.

#### Comparison of descriptive statistics for WISH, diet-related GHGE, and LU of observed vs. simulated data

The median (interquartile range, IQR) of the WISH score, diet-related GHGE, and LU were recalculated for each of the simulations created. The Mann–Whitney test was used to statistically compare the medians between the observed and the simulated datasets.

#### Potential impact fraction (PIF)

To estimate the potential reduction in the incidence of obesity that would result from each of the simulations created, the Potential Impact Fraction (PIF) for each individual was calculated using the following formula:

$$PIF=\frac{E\left[RR\left(X;\theta \right)\right]-\left[RR\left(cft(X);\theta \right)\right]}{\left[RR\left(X;\theta \right)\right]}$$

In this formula, $RR\left(X;\theta \right)$ represents the relative risk (RR) for each food group simulation under the baseline scenario, while $RR\left(cft(X);\theta \right)$ represents the RR for each food group simulation under the counterfactual scenario. The relative risk is calculated as follows:

$$RR=\mathrm{exp}\left(\mathrm{log}\left(a\right) x\right))$$

where *a* is the association between the WISH and obesity extracted from a previous study from the Generation XXI study [20] (estimation of the RR through the proxy Prevalence ratios = 0.912, 95%CI 0.839–0.99) and *x* is the score of the WISH for each individual. The number of prevented cases is given by

$$n=PIF x N x {I}_{p}$$

where N is number of individuals in the population and ${I}_{p}$ is the incidence of obesity in the population. The number of individuals in the population by age category was obtained from census data [21]. Data on obesity incidence and 95% CIs were obtained from the Generation XXI Portuguese birth cohort [22]. The median and respective confidence interval (CI) of the PIF and *n* for each scenario were calculated.

Differences in the consumption of food groups according to the established dietary patterns were assessed using the Kruskal–Wallis test. Analysis of variance (ANOVA) was used to examine differences in dietary patterns according to sex, exact age, parental education, and total energy intake. Bonferroni correction was applied to adjust for multiple testing, and statistical significance was defined as a *p*-value < 0.005. Statistical analyses were performed using R software (version 3.4.1 for Windows).

## Results

Participants had a similar distribution by sex. Obesity prevalence was approximately 10% in both children and adolescents. High parental education levels were observed in 45.7% of children and 32.1% of adolescents (Table 1).

**Table 1:** Characteristics of participants from the National Food, Nutrition and Physical Activity Survey, Portugal 2015–2016, stratified by age group: children (n = 521) and adolescents (n = 632)

	Children (3–9 years old)	Adolescents (10–17 years old)
Participant’s data		
Sex, n (%)		
Female	262 (50.3)	319 (50.5)
Male	259 (49.7)	313 (49.5)
Age, years, mean (SD)	5.9 (1.8)	13.3 (2.1)
BMI z-score ^a^ n (%)		
Normal and underweight	315 (68.5)	410 (66.2)
Preobese	96 (21.0)	143 (23.1)
Obese	46 (10.1)	66 (10.7)
Missings (n)	64	13
Parents’ data		
Mother’s age, years, mean (SD)	37.4 (5.1)	42.2 (5.3)
Father’s age, years**,** mean (SD)	39.9 (5.7)	45.4 (5.9)
Parent’s education considering the highest level, n (%)		
≤ 9th grade (≤ 9 years)	139 (26.7)	237 (37.5)
10th–12th grade (10–12 years)	143 (27.4)	182 (28.8)
Higher education (> 12 years)	238 (45.7)	203 (32.1)
Does not know/Prefer not to answer	1 (0.2)	10 (1.6)

BMI, Body Mass Index; n, sample size; SD, standard deviation

^a^Defined according to the World Health Organization (14,15)

Using a latent class model with no concomitant variables, three latent classes (dietary patterns, DPs) were identified for children and adolescents. Model selection was based on goodness-of-fit indices and interpretability, with the final decision supported by comparison of AIC and BIC values and the stability of the 3-class solution.

Tables 2 and 3 show the probabilities of food group consumption according to the DP derived in children and adolescents. In children, DP1 had a higher percentage of individuals in the upper category of intake (above the sample median) of red and processed meat, white meat, eggs, butter, and soft drinks (referred to from now on as “Meat, eggs and soft drinks”). DP2 was characterised by children with the highest intake of nuts, fruits, vegetables, legume grains, olive oil, pasta, rice, bread and toasts (referred to from now on as “Plant-based foods”). DP3 included children who typically exhibit average consumption of most foods compared to the other two patterns, although they consume more milk and less meat, fish, and soft drinks (referred to from now on as “Less Meat, Fish and Soft Drinks”) (Table 2). In adolescents, DP1 was distinguished by the highest uptake of nuts, cereals (pasta, rice, bread), olive oil, vegetables, fruits, and legume grains (referred to from now on as “Plant-based foods”). The other two DPs identified among adolescents (DP2 and DP3) were characterised by a higher percentage of individuals in the upper intake category of soft drinks. However, they differed in the type of animal-based products most consumed: one group exhibits the highest prevalence of consumption above the median of red meat, while the other predominantly consumes white meat and fish (referred to from now on as “Soft Drinks and Red Meat” and “Soft Drinks, Fish, and White Meat”, respectively) (Table 3). The median consumption of the 13 food groups considered in the WISH according to the DP is described in Supplementary Table C. The characterization of dietary patterns according to age, sex, parents’ education and energy is shown in Supplementary Table D.

**Table 2:** Probabilities of food group consumption conditional on a dietary pattern derived from the latent class model for children

		*DP1*	*DP2*	*DP3*		*DP1*	*DP2*	*DP3*
		Meat, eggs and soft drinks	Plant-based foods	Less meat, fish and soft drinks		Meat, eggs and soft drinks	Plant-based foods	Less meat, fish and soft drinks
Nil (%)	Pasta	39.0	25.3	16.8	Rice	23.9	18.7	26.7
≤ Median (%)		35.9	29.5	44.6		31.6	42.4	53.5
> Median (%)		25.1	**45.2**	38.6		**44.5**	38.9	19.8
Nil (%)	Bread and toasts	–	–	–	Other grains*	90.4	92.1	91.3
≤ Median (%)		52.8	43.4	81.8		–	–	–
> Median (%)		47.2	**56.6**	18.2		9.7	7.9	8.7
Nil (%)	Nuts	77.1	73.7	91.5	Seeds*	99.5	95.5	97.9
≤ Median (%)		13.2	9.7	8.5		–	–	–
> Median (%)		9.8	**16.6**	0.0		0.5	0.5	**2.1**
Nil (%)	Fresh fruit	–	–	–	Fresh vegetables	–	–	–
≤ Median (%)		68.3	35.1	56.9		81.9	12.5	51.5
> Median (%)		31.8	**64.9**	43.1		18.2	**87.5**	48.5
Nil (%)	Legume grains	64.1	43.1	36.1	Olive oil*	73.1	8.9	78.5
≤ Median (%)		20.0	21.4	41.9		–	–	–
> Median (%)		15.9	**35.5**	22.0		26.9	**91.1**	51.5
Nil (%)	Vegetable oils	29.2	38.5	51.7	Margarine	68.9	58.4	87.9
≤ Median (%)		31.9	30.7	33.2		20.3	24.0	12.1
> Median (%)		**39.0**	**30.8**	15.1		10.8	**17.6**	0.0
Nil (%)	Milk	–	–	–	Milk shake and flavored milk	63.0	60.7	78.8
≤ Median (%)		59.8	55.6	34.7		16.0	23.7	13.4
> Median (%)		40.2	44.4	**65.3**		**21.0**	15.6	7.7
Nil (%)	Yogurt	41.6	31.5	21.9	Cheese	46.1	35.6	42.4
≤ Median (%)		41.1	33.8	46.4		27.0	25.9	41.9
> Median (%)		17.3	**34.7**	31.8		26.9	**38.5**	15.7
Nil (%)	Red Meat	31.6	25.9	19.6	Processed meat	18.1	19.7	43.5
≤ Median (%)		32.2	34.7	50.3		36.2	36.3	47.8
> Median (%)		**39.4**	36.2	**30.1**		**45.7**	43.9	8.7
Nil (%)	White meat	34.1	35.3	25.0	Eggs	37.8	43.2	36.1
≤ Median (%)		25.5	30.6	58.9		24.8	31.0	41.9
> Median (%)		**40.5**	34.1	**16.2**		**37.4**	25.8	22.0
Nil (%)	Fresh Fish	50.5	34.4	46.8	Shellfish*	91.1	90.5	91.7
≤ Median (%)		25.9	24.0	42.4		–	–	–
> Median (%)		23.7	**41.7**	**10.9**		8.9	**9.6**	8.3
Nil (%)	Butter	43.2	49.8	87.9	Other saturated fats	88.2	86.3	82.3
≤ Median (%)		25.1	21.6	12.1		5.4	7.8	8.6
> Median (%)		**31.7**	28.7	**0.0**		6.4	6.0	9.1
Nil (%)	Soft drinks	30.3	59.8	70.3	Added sugar	61.1	66.0	78.8
≤ Median (%)		25.3	24.8	29.7		17.0	17.3	15.9
> Median (%)		**44.5**	15.4	**0.0**		**21.9**	16.7	**5.3**

*For these foods, where it is written “ > Median (%)”, it should be read as “consumes”, as the categorisation of these food groups was “consumes/does not consume (nil consumption)”

**Table 3:** Probabilities of food group consumption conditional on a dietary pattern derived from the latent class model for adolescents

		*DP1*	*DP2*	*DP3*		*DP1*	*DP2*	*DP3*
		Plant-based foods	Soft drinks and red meat	Soft drinks, fish, and white meat		Plant-based foods	Soft drinks and red meat	Soft drinks, fish, and white meat
Nil (%)	Pasta	30.19	34.79	43.73	Rice	24.15	24.23	39.81
≤ Median (%)		32.13	35.79	25.66		36.88	39.06	36.44
> Median (%)		**37.68**	29.42	30.61		**38.98**	36.71	23.75
Nil (%)	Bread and toasts	–	–	–	Other grains*	86.67	92.79	89.22
≤ Median (%)		49.12	56.29	60.00		–	–	–
> Median (%)		**50.88**	43.71	40.00		**13.33**	7.21	10.78
Nil (%)	Nuts	69.46	5.06	72.57	Seeds*	97.41	98.15	100.0
≤ Median (%)		15.27	7.53	14.80		–	–	–
> Median (%)		**15.27**	7.41	12.63		2.59	1.85	0.00
Nil (%)	Fresh fruit	10.38	30.68	34.30	Fresh vegetables	–	–	–
≤ Median (%)		39.41	38.36	33.72		0.00	77.60	72.79
> Median (%)		**50.21**	30.96	31.98		**100.0**	22.40	27.21
Nil (%)	Legume grains	43.47	61.68	74.80	Olive oil*	–	–	–
≤ Median (%)		23.65	23.27	11.09		19.45	60.80	79.56
> Median (%)		**32.88**	15.05	14.12		**80.55**	39.20	20.44
Nil (%)	Vegetable oils	38.36	32.63	48.62	Margarine	69.85	65.55	74.17
≤ Median (%)		33.41	34.10	21.79		14.05	17.50	14.02
> Median (%)		28.22	**33.27**	29.59		16.09	**16.95**	11.81
Nil (%)	Milk	9.40	49.17	41.43	Milk shake and flavored milk	78.50	83.73	76.34
≤ Median (%)		15.91	41.93	42.16		11.79	6.73	13.81
> Median (%)		17.01	38.30	**44.69**		9.71	9.53	9.85
Nil (%)	Yogurt	53.54	55.36	69.28	Cheese	27.23	44.17	35.99
≤ Median (%)		19.66	23.10	20.12		38.55	29.14	25.67
> Median (%)		**26.80**	21.54	10.60		34.22	26.70	**38.34**
Nil (%)	Red Meat	17.68	0.00	100.0	Processed meat	15.37	22.69	17.97
≤ Median (%)		42.33	49.15	0.00		42.60	41.19	34.39
> Median (%)		39.98	**50.85**	0.00		42.04	36.13	**47.64**
Nil (%)	White meat	40.36	40.77	25.41	Eggs	37.85	48.99	43.38
≤ Median (%)		33.91	34.46	26.59		28.03	27.24	29.81
> Median (%)		25.73	24.77	**47.99**		**34.11**	23.77	26.81
Nil (%)	Fresh Fish	44.08	59.55	43.70	Shellfish*	90.59	89.85	88.69
≤ Median (%)		30.13	20.88	22.76		3.17	5.65	7.77
> Median (%)		25.78	19.57	**33.53**		6.24	4.51	3.54
Nil (%)	Butter	45.66	48.27	54.41	Other saturated fats	87.77	75.40	100.0
≤ Median (%)		21.98	27.02	31.10		6.38	12.28	0.00
> Median (%)		**32.36**	24.71	14.50		5.85	**12.32**	0.00
Nil (%)	Soft drinks	38.42	30.46	32.50	Added sugar	51.83	63.95	66.97
≤ Median (%)		40.90	31.04	25.13		24.54	17.12	18.73
> Median (%)		20.67	**38.50**	**42.37**		**23.63**	18.93	14.30

*For these foods, where it is written “ > Median (%)”, it should be read as “consumes”, as the categorisation of these food groups was "consumes/does not consume (nil consumption)"

Table 4 presents the descriptives of the food group consumption in the observed and simulated scenarios. Firstly, it is noteworthy that in the observed (real) scenario, only the median consumption of fruit and fish among children and white meat among adolescents were aligned with the recommended targets (124.0 vs. 124.0 g/day, 16.1 vs. 17.0 g/day, and 27.6 vs. 29.0 g/day, respectively). In all other simulations, the target thresholds were not fully achieved while the simulated values approached the recommendations. For example, in children, dairy consumption decreased from a median of 469.7 g/day in the observed scenario to 415.2 g/day in the simulated one, with the target being 154 g/day. Similarly, legume grain consumption increased from a median of 0 g/day in the observed scenario to 5.3 g/day in the simulated scenario, still below the target of 46 g/day (Table 4).

**Table 4:** Descriptives of the food group consumption in the observed and simulated scenarios and probabilities of individuals being classified into each dietary pattern within the scenarios simulated for children and adolescents

	Observed Median (IQR), g/day	Recommended target, g/day*	Simulated Median (IQR), g/day	Latent Class 1	Latent Class 2	Latent Class 3
Children				Meat, eggs and soft drinks	Plant-based foods	Less meat, fish and soft drinks
Observed				44%	37%	19%
*Scenarios simulated*						
1 Dairy	469.7 (295.5)	154	415.2 (330.1)	98%	1%	1%
Saturated fats	1.9 (5.0)	7	1.9 (5.0)	98%	1%	1%
2 Vegetables	120.1 (101.3)	185	164.8 (81.6)	3%	91%	6%
Cereals	134.6 (94.4)	174	148.6 (92.1)	1%	98%	1%
Legume grains	0.0 (13.3)	46	5.3 (19.8)	1%	98%	1%
Nuts	0.0 (0.0)	31	0.0 (0.8)	1%	98%	1%
Unsaturated oils	10.7 (8.8)	25	13.7 (9.2)	1%	98%	1%
3 Fruits	124.0 (131.1)	124	110.4 (111.1)	53%	11%	36%
4 Eggs	2.7 (22.2)	8	1.0 (9.9)	5%	20%	76%
Fish and shellfish	16.1 (40.8)	17	8.7 (27.2)	21%	9%	71%
Chicken and other poultry	23.8 (56.9)	18	22.2 (34.3)	1%	1%	98%
Red and processed meat	45.6 (54.9)	9	24.1 (40.8)	1%	1%	98%
Soft drinks and added sugars	25.3 (139.4)	19	0.0 (34.0)	1%	1%	98%

Adolescents				Plant-based foods	Soft Drinks and Red Meat	Soft Drinks, Fish, and White Meat
Observed				34%	47%	19%
*Scenarios simulated*						
1 Vegetables	101.3 (98.2)	300	160.7 (72.6)	98%	1%	1%
Fruits	85.1 (145.9)	200	112.5 (151.6)	98%	1%	1%
Cereals	196.7 (148.3)	282	215.5 (168.6)	98%	1%	1%
Nuts	0.0 (0.0)	50	0.0 (1.9)	98%	1%	1%
Legume grains	0.0 (12.3)	75	4.9 (23.4)	98%	1%	1%
Unsaturated oils	12.0 (11.0)	40	14.9 (10.7)	98%	1%	1%
Saturated fats	2.5 (7.5)	11.8	5.0 (10.0)	98%	1%	1%
Soft drinks and added sugars	157.2 (335.6)	33	120.6 (210.7)	98%	1%	1%
2 Chicken and other poultry	27.6 (73.8)	29	27.2 (72.3)	1%	98%	1%
3 Eggs	3.4 (25.7)	13	2.7 (23.0)	6%	68%	27%
4 Fish and shellfish	9.4 (41.8)	28	10.4 (45.1)	35%	27%	38%
5 Red and processed meat	71.5 (85.5)	14	21.0 (37.4)	1%	1%	98%
Dairy	346.7 (280.3)	250	330.1 (328.9)	1%	1%	98%

IQR, interquartile range

*According to the adapted Eat-Lancet target values for children and adolescents (11)

A detailed description of the food groups is given in Supplementary Table A

Table 4 also shows the probabilities of individuals being classified into each DP within the scenarios simulated for children and adolescents. For example, in children, to align with the EAT-Lancet reference diet for eggs, fish, white meat, red and processed meat, and soft drinks and added sugars, a substantial shift towards the "Less Meat, Fish and Soft Drinks" DP is needed from a probability of 18.6% of individuals being classified in this DP in the real scenario to over 70% in the simulated scenario. In adolescents, in Scenario 1, there is a notable increase in the probability of individuals being classified into the “Plant-Based Foods” DP, from 33.8% in the observed data to 98.0% in the simulated data, to meet the EAT-Lancet reference diet for vegetables, fruits, cereals, nuts, legume grains, and both unsaturated and saturated fats.

Table 5 presents the effect of each food group simulation on the WISH score and diet-related GHGE and LU for both children and adolescents. Additionally, the table includes the WISH score, GHGE, and LU estimates for the observed (real) scenario in comparison to the simulated scenarios.

**Table 5:** Effect of each simulated scenario on the WISH score, diet-related GHGE and LU in children and adolescents

	WISH Median (IQR)	p-value*	GHGE Median (IQR), kg CO_2_ eq	p-value*	Land use Median (IQR), m^2^/year	p-value*
*Children*						
*Observed*	50.0 (21.0)		3.9 (1.5)		3.4 (1.4)	
*Scenarios simulated*						
1 Dairy	41.6 (20.6)	** < 0.001**	3.7 (1.7)	0.009	3.3 (1.7)	0.080
Saturated fat^s^	41.6 (20.6)	** < 0.001**	3.7 (1.7)	0.009	3.3 (1.7)	0.080
2 Vegetables	57.8 (19.8)	** < 0.001**	4.1 (1.8)	0.030	3.5 (1.6)	0.070
Cereals	59.6 (18.8)	** < 0.001**	4.1 (1.7)	0.030	3.5 (1.7)	0.070
Legume grains	59.6 (18.8)	** < 0.001**	4.1 (1.8)	0.030	3.5 (1.7)	0.070
Nuts	59.6 (18.8)	** < 0.001**	4.1 (1.8)	0.030	3.5 (1.7)	0.070
Unsaturated oils	59.6 (18.8)	** < 0.001**	4.1 (1.8)	0.030	3.5 (1.7)	0.070
3 Fruits	47.3 (20.5)	0.030	3.7 (1.8)	**0.001**	3.2 (1.7)	**0.001**
4 Eggs	53.3 (18.8)	** < 0.001**	3.5 (1.6)	** < 0.001**	3.0 (1.5)	** < 0.001**
Fish and shellfish	51.6 (19.6)	0.260	3.5 (1.7)	** < 0.001**	3.0 (1.5)	** < 0.001**
Chicken and other poultry	52.8 (18.0)	0.006	3.3 (1.6)	** < 0.001**	2.8 (1.4)	** < 0.001**
Red and processed meat	52.8 (18.0)	0.006	3.3 (1.6)	** < 0.001**	2.8 (1.4)	** < 0.001**
Soft drinks and added sugars	52.8 (18.0)	0.006	3.3 (1.6)	** < 0.001**	2.8 (1.4)	** < 0.001**
*Adolescents*						
*Observed*	45.7 (18.3)		4.5 (2.3)		4.1 (2.2)	
*Scenarios simulated*						
1 Vegetables	49.5 (18.5)	** < 0.001**	4.8 (2.3)	** < 0.001**	4.3 (2.2)	0.010
Fruit	48.7 (18.6)	**0.001**	4.8 (2.2)	** < 0.001**	4.4 (2.1)	**0.004**
Cereals	48.7 (18.6)	**0.001**	4.8 (2.2)	** < 0.001**	4.4 (2.1)	**0.004**
Nuts	48.7 (18.6)	**0.001**	4.8 (2.2)	** < 0.001**	4.4 (2.1)	**0.004**
Legume grains	48.7 (18.6)	**0.001**	4.8 (2.2)	** < 0.001**	4.4 (2.1)	**0.004**
Unsaturated oils	48.7 (18.6)	**0.001**	4.8 (2.2)	** < 0.001**	4.4 (2.1)	**0.004**
Saturated fats	48.7 (18.6)	**0.001**	4.8 (2.2)	** < 0.001**	4.4 (2.1)	**0.004**
Soft drinks and added sugars	48.7 (18.6)	**0.001**	4.8 (2.2)	** < 0.001**	4.4 (2.1)	**0.004**
2 Chicken and other poultry	41.5 (17.7)	** < 0.001**	4.7 (2.2)	0.050	4.3 (2.2)	0.007
3 Eggs	43.3 (16.7)	0.006	4.3 (2.1)	0.150	3.9 (2.0)	0.220
4 Fish and shellfish	45.3 (17.5)	0.82	4.2 (2.1)	**0.002**	3.8 (2.0)	**0.001**
5 Red and processed meat	45.6 (19.0)	0.65	3.4 (1.6)	** < 0.001**	3.0 (1.5)	** < 0.001**
Dairy	45.6 (19.0)	0.65	3.4 (1.6)	** < 0.001**	3.0 (1.5)	** < 0.001**

*Mann–Whitney test.

Bold values indicate statistical significance (p < 0.005)

According to the adapted Eat-Lancet target values for children and adolescents (11)

A detailed description of the food groups is given in Supplementary Table A

**Fig. 2:**
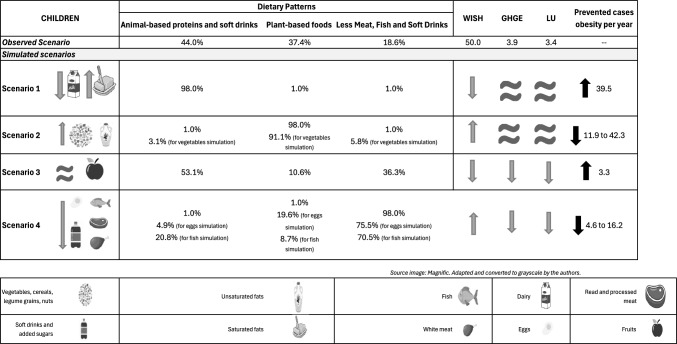
Summary of the main results regarding simulation modelling for children. Legend: GHGE: Green-house gas emission (Kg CO2 eq); LU: Land Use (m2/year); WISH: World Index for Sustainability and Health (points ranging from 0–130)

In children, Scenario 1 is particularly notable as it involves reducing dairy consumption to better align with the EAT-Lancet reference diet. This reduction resulted into a significant decrease in the WISH median score (from 50 points in the observed scenario to 41.6 points in the simulated scenario, p < 0.001) and the diet-related GHGE (from 3.9 kg CO_2_eq/day in the observed scenario to 3.7 kg CO_2_eq/day in the simulated scenario, p = 0.009). In contrast, Scenario 2 involves an increase in the consumption of vegetables, cereals, legume grains, nuts, and unsaturated fats to meet the target values, leading to a significant increase in both the WISH score and diet-related GHGE (from 3.9 kg CO_2_eq/day in the observed scenario to 4.1 kg CO_2_eq/day in the simulated scenario, p = 0.030). In Scenario 4, a reduction in the consumption of eggs, fish, white meat, red and processed meats, saturated fats, soft drinks, and added sugars led to a significant increase in the WISH score and the highest decrease in diet-related GHGE and LU (from 3.9 kg CO_2_eq/day and 3.4 m^2^/year in the observed scenario to 3.3 kg CO_2_eq/day and 2.8 m^2^/year in the simulated scenario, p < 0.001) (Table 5 and Fig. 2).

**Fig. 3:**
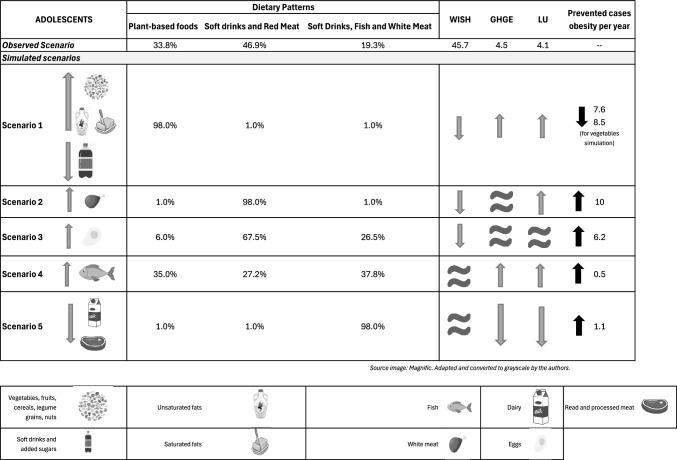
Summary of the main results regarding simulation modelling for adolescents. Legend: GHGE: Green-house gas emission (Kg CO2 eq); LU: Land Use (m2/year); WISH: World Index for Sustainability and Health (points ranging from 0–130)

In adolescents, Scenario 1 is noteworthy for the increased median consumption of vegetables, cereals, fruits, nuts, legume grains, and unsaturated and saturated fats, alongside a decrease in soft drinks and added sugars consumption. These changes led to a significant increase in both the WISH score and diet-related GHGE and LU. Conversely, in Scenario 5, characterised by reduced dairy and red meat intake to meet the target values, there was a significant decrease in diet-related GHGE and LU, while the WISH scores remained similar to the median observed in the real scenario (Table 5 and Fig. 3).

Table 6 presents the impact of each simulated scenario in preventing obesity cases by age group. For both children and adolescents, the potential impact fraction (PIF) was higher in the scenario involving an increase in the consumption of plant-based products to meet the target values (scenario 2 in children and 1 in adolescents), indicating that 0.7% and 0.2% of obesity cases in children and adolescents, respectively, could potentially be prevented if the proposed dietary changes were implemented. This corresponds to an estimated 12 to 42 cases prevented among children and approximately 8 cases among adolescents per year. Conversely, in children, Scenario 1, which involves a decrease in dairy and saturated fats consumption to meet the target value, is associated with the largest increase in obesity cases, with an estimation of almost 40 additional cases of obesity per year (PIF = − 0.7%). Among adolescents, all scenarios except Scenario 1 were associated with an increase in obesity cases, with the scenario decreasing white meat consumption (and transitioning to the “Soft drinks and Red meat” DP) showing the highest impact fraction of -0.3% and an increase of 10 obesity cases per year.

**Table 6:** Potential impact fraction (PIF) and prevented obesity cases due to the different scenarios created to achieve the targets of the EAT-Lancet reference diet in children and adolescents

	PIF (%) (95% CI)	Prevented obesity cases per year (95% CI)
*Children*		
Scenario 1		
Dairy	− 0.7 (− 0.8, − 0.6)	− 39.5 (− 43.3, − 35.5)
Saturated fats	− 0.7 (− 0.8, − 0.6)	− 39.5 (− 43.3, − 35.5)
Scenario 2		
Vegetables	0.7 (0.7, 0.8)	40.0 (36.2, 44.5)
Cereals	0.7 (0.7, 0.8)	11.9 (10.7, 12.9)
Legume grains	0.7 (0.7, 0.8)	42.3 (38.2, 46.0)
Nuts	0.7 (0.7, 0.8)	11.9 (10.7, 12.9)
Unsaturated oils	0.7 (0.7, 0.8)	42.3 (38.2, 46.0)
Scenario 3		
Fruits	− 0.2 (− 0.3, − 0.1)	− 3.3 (− 4.4, − 2.2)
Scenario 4		
Eggs	0.2 (0.2, 0.4)	16.2 (12.3, 20.1)
Fish and shellfish	0.1 (0.0, 0.2)	4.6 (0.8, 8.6)
Chicken and other poultry	0.2 (0.1, 0.3)	12.2 (8.5, 16.3)
Red and procesed meat	0.2 (0.1, 0.3)	12.2 (8.5, 16.3)
Soft drinks and added sugars	0.2 (0.1, 0.3)	12.2 (8.5, 16.3)
*Adolescents*		
Scenario 1		
Vegetables	0.3 (0.2, 0.3)	8.5 (6.4, 10.6)
Fruits	0.2 (0.2, 0.3)	7.6 (5.4, 9.6)
Cereals	0.2 (0.2, 0.3)	7.6 (5.4, 9.6)
Nuts	0.2 (0.2, 0.3)	7.6 (5.4, 9.6)
Legume grains	0.2 (0.2, 0.3)	7.6 (5.4, 9.6)
Unsaturated oils	0.2 (0.2, 0.3)	7.6 (5.4, 9.6)
Saturated fats	0.2 (0.2, 0.3)	7.6 (5.4, 9.6)
Soft drinks and added sugars	0.2 (0.2, 0.3)	7.6 (5.4, 9.6)
Scenario 2		
Chicken and other poultry	− 0.3 (− 0.4, − 0.3)	− 10.0 (− 11.9, − 8.1)
Scenario 3		
Eggs	− 0.2 (− 0.3, − 0.1)	− 6.2 (− 8.2, − 4.2)
Scenario 4		
Fish and shellfish	− 0.01 (− 0.1, 0.1)	− 0.5 (− 2.6, 1.8)
Scenario 5		
Red and processed meat	− 0.03 (− 0.1, 0.0)	− 1.1 (− 3.1, 1.2)
Dairy	− 0.03 (− 0.1, 0.0)	− 1.1 (− 3.1, 1.2)

CI, confidence interval; PIF, potential impact fraction

*A detailed description of the food groups is given in Supplementary Table A

The transitions between DPs, and the impact on WISH scores, GHGE, LU, and obesity cases prevented in each scenario are illustrated in Figs. 2 (children) and 3 (adolescents).

## Discussion

The study identified distinct DP in children and adolescents and modelled several realistic simulations of transitions to align with the EAT-Lancet reference diet, showing changes in health and environmental outcomes. Among children, the scenario involving the reduction of eggs, fish, white, red and processed meats, soft drinks, and added sugars (scenario 4) led to a significant increase in the WISH scores, alongside the most significant decrease in diet-related GHGE and LU. In adolescents, reducing red and processed meats and dairy (scenario 5) was particularly noteworthy, as it led to the most significant decrease in diet-related GHGE and LU. At the same time, WISH scores remained consistent with the median observed in the real scenario. However, the greatest impact in obesity-prevented cases was observed in the scenario related to a potential increase of plant-based foods in children and adolescents. Nevertheless, in these scenarios, there was also an increase in environmental indicators, potentially driven by a simultaneous increase in the consumption of animal-based foods during dietary transitions. These findings highlight that improvements in dietary quality indicators do not always align with the largest reductions in environmental impacts, consistent with previous studies [23, 24].

The EAT-Lancet reference diet adapted for children and adolescents [8] are extremely difficult to achieve, given the substantial gap between current dietary habits and the recommended targets (e.g., Portuguese adolescents consume an average of 1.7 g/day of nuts [25], compared to the recommended 50 g/day). We employed a novel approach to address this challenge by creating dietary patterns using latent classes models rather than using strict cut-off values from the recommendations. This method offers a key advantage, as it allows for a more realistic assessment of achieving dietary goals by simulating changes/transitions in overall dietary patterns rather than individual food groups, as is typically done in single-food scenarios. By considering shifts between dietary patterns, this approach reflects more practical and attainable efforts to meet the dietary targets. It provides a more realistic simulation of how individuals might adjust their consumption to maximise adherence to the recommended guidelines. These transitions are, therefore, more realistic and based on well-fitted dietary scenarios. To our knowledge, this is the first study to perform such analysis, deriving a posteriori dietary pattern based on the food groups recommended by the EAT-Lancet Commission [7].

Dietary patterns characterised by plant-based foods were identified in children and adolescents. However, the results show that the median consumption of these foods remains far below the EAT-Lancet dietary recommendations adapted for paediatric ages [8]. Notably, only the median of fruit and fish consumption in children and white meat consumption in adolescents came close to the recommended levels. This discrepancy underscores a considerable gap between current dietary habits and the optimal targets established by the EAT-Lancet recommendations, highlighting the challenges of meeting these nutritional guidelines.

From a health perspective, particularly considering the calculation of population impact fractions for obesity prevention, the scenario promoting an increase in the consumption of plant-based foods seems to be the most effective for children and adolescents. This scenario could potentially prevent the most significant number of obesity cases annually but in a small magnitude (maximum of 42 cases per year). This underscores the relative importance of plant-based diets in reducing obesity among younger populations [26, 27].

On the other hand, simulating scenarios that closely align with the EAT-Lancet reference diet for plant-based foods resulted in nearly all individuals (98%) shifting to the "Plant-based foods" dietary pattern in both children and adolescents. Upon examining the profile of this dietary pattern (Supplementary Table C), we observe that while it emphasises a higher consumption of plant-based foods, it still includes a modest consumption of dairy, eggs, fish, red meat, and soft drinks. As a result, this broader rise in food consumption could lead to greater environmental impacts, such as higher diet-related GHGE and LU. Therefore, significantly increasing plant-based food consumption may not be feasible without exacerbating environmental burdens, highlighting the complexity of balancing health and sustainability goals in dietary strategies. Although adherence to dietary guidelines is generally associated with lower environmental impacts compared with business-as-usual diets, our findings suggest that this relationship may not be straightforward and can depend on baseline dietary intake. In particular, improving dietary adequacy to better meet nutritional requirements may, in some contexts, be associated with increases in environmental impacts depending on the initial diet composition.

In children, the scenario that best balances health and environmental sustainability involves reducing the intake of eggs, fish, white and red meat, and soft drinks and shifting to the “Less Meat, Fish and Soft drinks” pattern. Reducing animal-based products and soft drinks likely decreases total energy intake (total food amounts) and particularly minimises the consumption of foods associated with higher GHGE and LU [28]. Animal agriculture, especially red meat production, is resource-intensive and environmentally taxing [29]. By reducing reliance on these foods, this scenario lowers environmental impact and promotes healthier diets. However, the potential implications of these dietary shifts for micronutrient adequacy were not specifically assessed and should be considered a limitation of this analysis.

Furthermore, reducing these foods (animal-based products and soft drinks) may naturally shift children’s diets towards lower energy diets, which are generally associated with lower environmental costs and better health outcomes [30]. This may suggest that the amount of food consumed may be as important as the specific choice of foods. In fact, the DP “Less Meat, Fish, and Soft Drinks” is also characterized by a generally intermediate consumption of most foods compared to the other two patterns (apart from those food groups highlighted in its label). This transition likely contributed to the improved WISH scores and reduced obesity cases observed in this scenario. Promoting moderate consumption across all food groups appears to be a more balanced solution for achieving health and environmental goals. However, practical implementation requires careful planning to ensure children still receive adequate nutrition, particularly in terms of protein, vitamins, and minerals typically supplied by the food groups aimed to decrease (i.e. animal protein-based foods). Importantly, any reduction in energy intake must be carefully monitored to avoid inadequacy, particularly in children, to ensure that overall energy requirements for growth and development are met.

The scenario that most reduced diet-related GHGE and LU for adolescents involves decreasing dairy and red meat consumption. While beneficial for environmental sustainability, this change did not significantly improve the WISH score and even increased the number of obesity cases by about one per year. This could be due to a shift towards a diet higher in fish and white meats, according to the scenarios simulated in this study, which, despite lowering environmental indicators, may not enhance overall diet quality as measured by the WISH score. The score considers a balanced intake of various food groups, including plant-based foods, not just the reduction of certain animal products [8]. These results highlight that reductions in specific animal-based food groups do not necessarily translate into an increased contribution of plant-based dietary patterns, as dietary changes are driven by complex substitutions across food groups rather than simple animal-to-plant dietary transitions. A similar pattern was observed in children in this study, where reducing dairy (scenario 1) but transitioning to the “Meat, eggs and soft drinks” and not to the “Plant-based foods” led to decreased GHGE but also worsened WISH scores and an increase in annual obesity cases. Therefore, only replacing dairy with other animal proteins without increasing nutrient-dense plant-based foods may not sufficiently improve diet quality and prevent obesity cases [31].

Overall, these findings highlight the complexity of achieving a balance between health and environmental goals, as already reported in the literature [3, 4]. While certain dietary changes may improve diet quality, they may simultaneously have adverse effects on environmental sustainability, and vice versa. This trade-off has also been documented in paediatric populations: van de Locht et al. [9] found that higher nutrient adequacy in children and adolescents was positively associated with greater GHGE and land use, underscoring that nutritionally richer diets do not necessarily translate into lower environmental impact diets in this age group. Although plant-based foods are generally associated with better health and environmental outcomes [26, 27], the relationship is not always straightforward when comprehensively assessed by dietary patterns, considering the interaction and cumulative effects of several foods and nutrients. Simply increasing plant-based food consumption or focusing solely on reducing animal-source foods without addressing overall dietary balance may not maximise health benefits or environmental sustainability.Therefore, public health messaging must carefully consider these trade-offs. For children, the emphasis should be on reducing the intake of animal-based products and soft drinks to a dietary pattern with an intermediate consumption of most food groups, which seems to align with health and environmental sustainability goals. Promoting plant-based diets may offer adolescents the greatest health benefits, particularly for obesity prevention. Still, it is essential also to consider strategies to mitigate the environmental impacts associated with increased plant-based consumption, such as reducing the consumption of dairy and red meat. Educating caregivers and young people about sustainable dietary practices through targeted health education programs can foster long-term behaviour change, supporting both individual and societal goals.

This study has several strengths and limitations that must be considered. First, the labelling of dietary patterns could introduce subjectivity, as evidenced by the plant-based pattern, which showed a median red meat consumption comparable to the “Meat, eggs and soft drinks” DP and substantially higher than the “Less Meat, Fish and Soft Drinks” DP (Supplementary Table C). This highlights the potential for interpretative bias in the results and a need of a comprehensive analysis of each DP.

Furthermore, the independent simulation of dietary patterns for each food group of the EAT-Lancet Commission, although methodologically justified, may limit the understanding of the interplay between groups. Employing an integrated modelling approach that considers all groups simultaneously could provide additional insights but would need important decisions on the weighting of each food group. Although analyses were stratified by age, the wide age range within the same group (such as 3 to 9 years old), cannot discard potential residual confounding. However, an examination of the mean ages of participants in each of the identified dietary patterns suggests overlap in age distributions across dietary patterns (Supplementary Table D). This overlap in covariance may mitigate the potential impact of age-related confounding, as the variation within groups is less pronounced than initially anticipated. Also, a key limitation of this study is the absence of a nutrient-level analysis, which limits the assessment of the potential nutritional consequences of the simulated dietary changes. Lastly, the use of BMI to classify obesity as the sole health indicator may not fully capture the complexity of health outcomes associated with dietary patterns. Despite these limitations, the statistical methods used in this study were highly innovative and robust, enabling a comprehensive analysis of complex dietary data from a representative sample of the Portuguese population.

### Conclusions

This study highlights specific realistic dietary scenarios aligned with the EAT-Lancet reference diet and evaluates their impact on diet-related GHGE, LU, and the obesity status of children and adolescents. For children, reducing the intake of eggs, fish, meat, and soft drinks led to the highest improvements in WISH scores and reductions in diet-related GHGE and LU. However, it was not associated with a decrease in obesity cases. In adolescents, while reducing dairy and red meat resulted in the greatest environmental benefits, it did not improve WISH scores nor prevent obesity cases. Notably, transitioning towards “Plant-based foods” dietary patterns was associated with the largest potential decrease in obesity cases in both age groups. Still, it also produced increases in environmental indicators, possibly driven by a concurrent rise in animal-based food consumption during these dietary shifts. In conclusion, promoting overall diet quality through balanced consumption of diverse food groups may be more effective than focusing solely on restricting/promoting specific foods, emphasising the importance of more comprehensive approaches for achieving health and environmental goals.

Public health recommendations should focus on promoting more balanced diets, incorporating greater consumption of plant-based foods while reducing excessive intakes of resource-intensive animal-source products to improve health outcomes and environmental sustainability. Further research is needed to refine these strategies and ensure their effective implementation across diverse populations.

## Supplementary Information

Below is the link to the electronic supplementary material.

Supplementary file1

## Data Availability

Data described in the manuscript, code book, and analytic code will be made available upon request pending.
