# Supplementary material for: Modelling EAT-lancet dietary patterns scenarios: effects on environmental and health metrics in children and adolescents
Source: Eur J Nutr. 2026 Jul 21;65(5):218. doi: 10.1007/s00394-026-04069-6 (PMC13388387; doi:10.1007/s00394-026-04069-6)
Supplement: Supplementary file 1 — Supplementary file1 [file 394_2026_4069_MOESM1_ESM.docx]

**Supplementary Table A: World Index for Sustainability and Health (WISH) components adapted to children and adolescents.**

| WISH food groups* | Components | Adaptation for children  3-9 years old (1544 kcal) | Adaptation for adolescents  10-17 years old (2549 kcal) | Maximum Score | Minimum Score |
| --- | --- | --- | --- | --- | --- |
|  |  | Recommended intake in g/day (lower and upper range of intake) ** | | **10 points**** | **0 points**** |
| Cereals (g/day) | pasta, rice, bread and toasts | 174 (139-209) | 282 (226 - 338) | :  $10 x \frac{reported intake lower recommended intake}{recommended intake lower recommended intake}$ | |
| Vegetables (g/day) | fresh, frozen, cooked, canned, or  dried vegetables, excluding salted or pickled vegetables | 185 (124-371) | 300 (200-600) |  |  |
| Fruits (g/day) | fresh, frozen, cooked, canned, or dried but excluding fruit juices, salted or pickled fruits | 124 (62-185) | 200 (100-300) |  |  |
| Legume grains (g/day) | fresh, frozen, cooked, canned, or dried legumes grains such as beans and lentils | 46 (0-62) | 75 (0-100) |  |  |
| Nuts (g/day) | tree nuts and ground nuts (including  peanuts) | 31 (0-46) | 50 (0-75) |  |  |
| Unsaturated oils(g/day) | olive, soybean, rapeseed, sunflower and peanut oil | 25 (12-49) | 40 (20-80) |  |  |
| Dairy (g/day) | whole or skimmed milk or derivative equivalents (e.g., cheese, yoghurt), excluding butter and cream | 154 (0-309) | 250 (0-500) |  |  |
| Fish and shellfish (g/day) | fish and shellfish | 17 (0-62) | 28 (0-100) |  |  |
| Chicken and other poultry (g/day) | chicken, duck, geese, rabbit | 18 (0-36) | 29 (0-58) | $10 x \frac{(upper recommended intake recommended intake) (reported intake recommended intake)}{upper recommended intake recommended intake}$ | |
| Eggs (g/day) | eggs from chickens and ducks, but excludes fish eggs | 8 (0-15) | 13 (0-25) |  |  |
| Red and processed meat (g/day) | beef, pork, lamb, and goat including red meat in its processed form but excluding poultry, fish, eggs | 9 (0-17) | 14 (0-28) |  |  |
| Saturated fats (g/day) | palm oil, and from dairy fats, lard or tallows | 7 (0-7) | 11.8 (0-11.8) | ≤ recommended intake | >recommended intake |
| Soft drinks and added sugars (g/day) | sugar-sweetened beverages and foods typically contributing to free sugars intake (e.g., sugar, honey, jams and similar products) | 19 (0-19) | 31 (0-31) | ≤ recommended intake | >recommended intake |

WISH: World Index for Sustainability and Health, originally described (12), and adapted to the pediatric age (11).

*For the calculation of the total WISH score, all components are summed up and are given equal weight in the total score. Possible score ranging from 0 to 130.

^**^ according to the European Food Safety Authority (EFSA) guidelines, Population Reference Intakes (PRI).

**Supplementary Table B.** Fit statistics from the latent class analysis models to derive dietary patterns in children (n=521) and adolescents (n=632).

|  | **AIC** | **p-value** | **BIC** | **p-value** |
| --- | --- | --- | --- | --- |
| **Children** | | | | |
| Number of latent classes |  |  |  |  |
| 1 | 23002.88 |  | **23194.38** |  |
| 2 | 22862.33 | <0.001 | 23249.61 | <0.001 |
| 3 | 22776.72 | <0.001 | 23359.76 | <0.001 |
| 4 | **22712.79** | <0.001 | 23491.59 | <0.001 |
| **Adolescents** | | | | |
| Number of latent classes |  |  |  |  |
| 1 | 28540.75 |  | **28754.30** |  |
| 2 | 28404.99 | <0.001 | 28840.53 | <0.001 |
| 3 | 28333.74 | <0.001 | 28983.28 | <0.001 |
| 4 | **28291.09** | <0.001 | 29158.63 | <0.001 |

Legend: AIC: Akaike information criterion; BIC: Bayesian information criterion

**Supplementary Table C.** Characterization based on food group consumption of the latent classes (dietary patterns) derived from the latent class models for children and adolescents.

|  | **Latent Class 1** | **Latent Class 2** | **Latent Class 3** |  |
| --- | --- | --- | --- | --- |
| *Children* |  | | |  |
|  | ***Meat, eggs and soft drinks*** | ***Plant-based foods*** | ***Less Meat, Fish and Soft Drinks*** | p-value* |
| ***Food groups*** | Median (IQR), g/day | | |  |
| Vegetables | 80.6 (56.2) | 182.0 (92.2) | 121.0 (76.4) | **<0.001** |
| Cereals | 138.0 (91.1) | 153.0 (99.4) | 101.0 (62.2) | **<0.001** |
| Legume grains | 0.0 (7.1) | 5.8 (18.2) | 4.9 (13.4) | **<0.001** |
| Nuts | 0.0 (0.0) | 0.0 (1.1) | 0.0 (0.0) | **0.001** |
| Fruits | 99.4 (106.0) | 167.0 (128.0) | 123.0 (104.0) | **<0.001** |
| Unsaturated oils | 8.8 (7.4) | 14.8 (7.6) | 6.5 (4.4) | **<0.001** |
| Dairy | 441.0 (288.0) | 472.0 (283.0) | 533.0 (254.0) | **0.001** |
| Eggs | 5.5 (29.6) | 3.0 (19.6) | 0.0 (5.7) | **0.001** |
| Fish and shellfish | 7.9 (20.2) | 27.8 (40.3) | 7.6 (16.5) | **0.001** |
| Chicken and other poultry | 23.2 (60.8) | 29.2 (59.0) | 18.3 (31.1) | 0.178 |
| Red meat | 50.8 (67.5) | 47.6 (53.0) | 26.3 (41.6) | **<0.001** |
| Saturated fats | 2.1 (5.7) | 1.3 (5.0) | 2.5 (4.1) | 0.196 |
| Soft drinks and added sugars | 115.0 (274.0) | 5.2 (101.0) | 0.0 (17.1) | **<0.001** |
| *Adolescents* |  | | |  |
|  | ***Plant-based foods*** | ***Soft Drinks and Red Meat*** | ***Soft Drinks, Fish, and White Meat*** |  |
| ***Food groups*** | Median (IQR), g/day | | |  |
| Vegetables | 178.0 (76.9) | 72.4 (59.3) | 71.1 (63.2) | **<0.001** |
| Cereals | 219.0 (160.0) | 203.0 (149.0) | 168.0 (126.0) | **<0.001** |
| Legume grains | 6.5 (24.8) | 0.0 (8.6) | 0.0 (0.9) | **<0.001** |
| Nuts | 0.0 (2.4) | 0.0 (0.0) | 0.0 (0.7) | **<0.001** |
| Fruits | 117.0 (151.0) | 55.4 (139.0) | 55.4 (139.0) | **<0.001** |
| Unsaturated oils | 15.0 (11.5) | 10.4 (10.1) | 8.6 (10.0) | **<0.001** |
| Dairy | 360.0 (254.0) | 338.0 (294.0) | 352.0 (289.0) | 0.091 |
| Eggs | 6.5 (30.4) | 0.5 (18.3) | 5.2 (22.0) | 0.003 |
| Fish and shellfish | 14.6 (20.3) | 0.0 (30.4) | 18.4 (14.9) | 0.047 |
| Chicken and other poultry | 24.4 (65.3) | 19.9 (60.8) | 58.8 (109.0) | 0.049 |
| Red meat | 74.6 (86.8) | 94.4 (90.0) | 23.0 (39.4) | **<0.001** |
| Saturated fats | 2.5 (10.0) | 2.5 (7.4) | 0.0 (5.0) | **<0.001** |
| Soft drinks and added sugars | 101.0 (220.0) | 172.0 (371.0) | 209.0 (394.0) | **<0.001** |

IQR: Interquartile Range

*Kruskall-Wallis test

**Supplementary Table D.** Characterization of the dietary patterns of children and adolescents according to age, gender and energy intake.

|  | **Latent Class 1** | **Latent Class 2** | **Latent Class 3** | **p-value*** |
| --- | --- | --- | --- | --- |
| *Children* |  |  |  |  |
|  | ***Meat, eggs and soft drinks*** | ***Plant-based foods*** | ***Less Meat, Fish and Soft Drinks*** |  |
| Sex, n (%) |  |  |  | 0.845 |
| Female | 116 (50.4) | 96 (49.0) | 50 (52.6) |  |
| Male | 114 (49.6) | 100 (51.0) | 45 (47.4) |  |
| Parent’s education considering the highest level, n (%) |  |  |  |  |
| ≤ 9th grade | 72 (31.3) | 34 (17.3) | 33 (34.7) | <0.001 |
| 10th – 12th grade | 70 (30.4) | 52 (26.5) | 21 (22.1) |  |
| >12 years | 88 (38.3) | 110 (56.1) | 40 (42.1) |  |
| Does not know/Prefer not to answer | 0 (0.0) | 0 (0.0) | 1 (1.1) |  |
| Age, mean (SD) | 6.2 (1.7) | 5.9 (1.8) | 4.8 (1.8) | <0.001 |
| Total energy intake (kcal/day), mean (SD) | 1659 (429.0) | 1749 (375.0) | 1366 (320.0) | <0.001 |
| *Adolescents* | ***Plant-based foods*** | ***Soft Drinks and Red Meat*** | ***Soft Drinks, Fish, and White Meat*** |  |
| Sex, n (%) |  |  |  |  |
| Female | 134 (49.3) | 71 (52.2) | 114 (50.9) | 0,845 |
| Male | 138 (50.7) | 65 (47.8) | 110 (49.1) |  |
| Parent’s education considering the highest level, n (%) |  |  |  |  |
| ≤ 9th grade (≤ 9 years) | 98 (36.0) | 45 (33.1) | 94 (42.0) | 0.308 |
| 10th – 12th grade (10-12 years) | 75 (27.6) | 46 /33.8) | 61 (27.3) |  |
| Higher education (>12 years) | 95 (34.9) | 42 (30.9) | 66 (29.5) |  |
| Does not know/Prefer not to answer | 4 (1.5) | 3 (2.2) | 3 (1.3) |  |
| Age, mean (SD) | 13.3 (2.1) | 13.2 (2.2) | 13.2 (2.1) | 0.768 |
| Total energy intake (kcal/day), mean (SD) | 2016 (596.0) | 1754 (520.0) | 2119 (600.0) | 0.978 |

SD: standard deviation

*ANOVA-test
